# Interim analysis of survival in a prospective, multi-center registry cohort of cutaneous melanoma tested with a prognostic 31-gene expression profile test

**DOI:** 10.1186/s13045-017-0520-1

**Published:** 2017-08-29

**Authors:** Eddy C. Hsueh, James R. DeBloom, Jonathan Lee, Jeffrey J. Sussman, Kyle R. Covington, Brooke Middlebrook, Clare Johnson, Robert W. Cook, Craig L. Slingluff, Kelly M. McMasters

**Affiliations:** 1 Dept. of Surgery, St. Louis University, St. Louis, MO USA; 2 South Carolina Skin Cancer Center, Greenville, SC USA; 3 Northside Melanoma and Sarcoma Specialists of Georgia, Atlanta, GA USA; 4 Dept. of Surgery, University of Cincinnati Cancer Institute, Cincinnati, OH USA; 5 Castle Biosciences, Inc., 820 S. Friendswood Drive Suite 201, Friendswood, TX USA; 6 Dept. of Surgery and Cancer Center, University of Virginia School of Medicine, Charlottesville, VA USA; 7 Dept. of Surgical Oncology, James Graham Brown Cancer Center, University of Louisville School of Medicine, Louisville, KY USA

**Keywords:** Gene expression profiling, DecisionDx-Melanoma, Cutaneous melanoma, Metastasis, Prognosis, Staging

## Abstract

**Background:**

A 31-gene expression profile (GEP) test that provides risk classification of cutaneous melanoma (CM) patients has been validated in several retrospective studies. The objective of the reported study was a prospective evaluation of the GEP performance in patients enrolled in two clinical registries.

**Methods:**

Three-hundred twenty two CM patients enrolled in the EXPAND (NCT02355587) and INTEGRATE (NCT02355574) registries met the criteria of age ≥ 16 years, successful GEP result and ≥1 follow-up visit for inclusion in this interim analysis. Primary endpoints were recurrence-free (RFS), distant metastasis-free (DMFS), and overall survival (OS).

**Results:**

Median follow-up was 1.5 years for event-free patients. Median age for subjects was 58 years (range 18–87) and median Breslow thickness was 1.2 mm (range 0.2–12.0). Eighty-eight percent (282/322) of cases had stage I/II disease and 74% (237/322) had a SLN biopsy. Seventy-seven percent (248/322) had class 1 molecular profiles. 1.5-year RFS, DMFS, and OS rates were 97 vs. 77%, 99 vs. 89%, and 99 vs. 92% for class 1 vs. class 2, respectively (*p* < 0.0001 for each). Multivariate Cox regression showed Breslow thickness, mitotic rate, and GEP class to significantly predict recurrence (*p* < 0.01), while tumor thickness was the only significant predictor of distant metastasis and overall survival in this interim analysis.

**Conclusions:**

Interim analysis of patient outcomes from a combined prospective cohort supports the 31-gene GEP’s ability to stratify early-stage CM patients into two groups with significantly different metastatic risk. RFS outcomes in this real-world cohort are consistent with previously published analyses with retrospective specimens. GEP testing complements current clinicopathologic features and increases identification of high-risk patients.

**Trial registration:**

ClinicalTrials.gov, NCT02355574 and NCT02355587

**Electronic supplementary material:**

The online version of this article (10.1186/s13045-017-0520-1) contains supplementary material, which is available to authorized users.

## Background

The majority of cutaneous melanoma (CM) patients are diagnosed with early-stage (AJCC stage I or II) disease and are considered to have a favorable prognosis [1, 2]. However, two-thirds of all melanoma deaths occur in patients initially included in this “low-risk” group, which indicates that metastatic risk is underestimated for a substantial number of early-stage patients [1–4]. A recent study reporting data from the Surveillance, Epidemiology, and End Results (SEER) program reported that the mortality rate for cutaneous melanoma is increasing faster than the incidence rate, highlighting the need for additional prognostic tools to supplement standard clinicopathologic factors and improve identification of high-risk disease [5]. More vigilant follow-up for high-risk melanoma patients, including the addition of imaging and increasing clinical assessment frequency, leads to earlier detection of asymptomatic distant metastatic disease, when tumor burden is lower, and when surgical approaches as well as contemporary therapies have greater benefit [5–11]. Distinguishing those CM patients with high-risk tumor biology from those who are categorized as low risk by TNM staging alone is therefore a clinically important goal.

A prognostic gene expression profile (GEP) test (DecisionDx-Melanoma, Castle Biosciences, Inc.) that evaluates 31 gene targets expressed in the primary melanoma tumor to provide a binary classification of low (class 1) or high (class 2) risk of metastasis within 5 years of the initial diagnosis has been previously reported [12]. The test assesses the expression of three control genes, four genes with proven prognostic utility for uveal melanoma tumors [13], and 24 gene targets previously reported to be differentially expressed in metastatic compared to primary tumors [14–20]. Performance of the test has been evaluated in several retrospective validation studies, showing that it accurately prognosticates survival independent of clinical or pathologic assessment alone [12, 21, 22], and though it was originally developed to identify the distant metastasis risk associated with stage I and II melanoma tumors, the prognostic capability of the test has recently been reported in patients with stage III disease. In order to prospectively evaluate outcomes for a real-world representative cohort of CM patients, two multi-center registry studies (EXPAND and INTEGRATE) were initiated. Here, we present an interim analysis of the survival outcomes for 322 patients enrolled in these registries.

## Methods

### Patients

Patients were enrolled in one of two prospective studies, EXPAND and INTEGRATE (Clinicaltrials.gov identifiers: NCT02355587 and NCT02355574). The protocols have identical enrollment criteria and aims, including (1) the tracking of outcomes of patients for whom GEP testing was completed, and (2) documentation of the clinical application of test results. The studies differ only in clinical data collection requirements based upon the administrative capabilities of the contributing centers. While a combined analysis of the two studies was originally planned based upon the similarity of protocols and aims, an interim analysis of this cohort was not included in the initial study design. Based upon the 1.1-year median time to recurrence observed for class 2 patients in previous validation studies, an unplanned interim analysis of this combined cohort of patients is warranted and expected to answer the hypothesis that class 1 and class 2 risk groups will have significantly different recurrence-free (RFS), distant metastasis-free (DMFS), and overall survival (OS) rates [12, 21, 22].

Eleven US dermatologic and surgical centers participated after receiving protocol approval from their Institutional Review Boards. The number and diversity of centers were selected to reduce bias arising from single centers. Physicians enrolled patients with a diagnosis of cutaneous melanoma who were ≥16 years of age and had a successful GEP test result. The GEP test was performed in a central CAP/CLIA laboratory using standard, published protocols as previously described [12].

Sample size calculations were originally developed from survival outcomes observed in a limited, retrospective analysis of the GEP test, with assumptions of 0.05 for alpha and a stringent power of 0.95. Based upon the rapid, significant separation of risk profiles for class 1 and class 2 cohorts reported in expanded analyses of the test since the inception of the studies (January, 2014), recruitment was discontinued prior to accrual of the patient numbers specified within the protocols. Clinical follow-up will continue through 5-year post-diagnosis for each patient (*n* = 322).

At the time of the initial patient evaluation, prior to GEP testing, the treating physician assessed each patient’s baseline data, including Breslow thickness (BT), ulceration status, T stage and mitotic rate. The majority of centers reported mitotic rate in mm^2^; for those that did not, high-powered fields (HPF) were converted to mm^2^ according to a conversion rate of 4 HPF per mm^2^ specified by the AJCC v7 guidelines [1, 23]. BT and mitotic rate were used as continuous variables while ulceration, SLN status and GEP class result were treated as categorical variables. All data were entered into a secure case report form and were abstracted and assembled independently of statistical analysis. Last censor date for clinical data was December 30, 2016.

### Statistical analyses

Accuracy of the test was measured by sensitivity or specificity. The former was measured as the number of cases with an event [recurrence (including in transit and regional nodal recurrences), distant metastasis or death] that were identified as class 2, while specificity was the number of cases that did not have a﻿ documented event that were﻿ classified as low-risk class 1 that did not have a documented event. Primary survival endpoints of RFS (time to regional or distant metastasis), DMFS (time to any metastatic event beyond the regional nodal basin), and OS (time from diagnosis to documented death of any cause) were assessed using Kaplan-Meier and Cox regression analyses. For multivariate analyses, only complete cases were considered. In 26 cases, ulceration, BT and/or mitotic rate were not known or specified and these cases were excluded from multivariate analyses. Cases without a documented SLN biopsy result were categorized as clinically node-negative.

As an interim analysis at year 3 of an expected 5-year study, the critical alpha level (*p* value) was established at 0.01. Statistical associations were evaluated using the *F* test, chi-squared test, *T* test, and ANOVA, where appropriate. Statistical analyses were performed in R version 3.3.2 (University of Auckland, NZ).

## Results

### Clinical and demographical comparison between GEP classes

A total of 335 patients were enrolled in the studies at the last censor date. Of these, 322 patients had completed at least one follow-up visit and were available for inclusion in this interim analysis. A GEP class 1 profile was observed in 77% (248 of 322) of samples. Table 1 provides demographic variables for the entire cohort, stratified by GEP class. The median age of subjects was 58 years (range 18–87 years) and the median Breslow thickness was 1.2 mm (range 0.2–12.0 mm). Within the group, 88% (282 of 322) of the cases had stage I or II disease, and SLN biopsy was performed for 74% (237 of 322) of the subjects. A class 2 profile was observed in 23% (74 of 322) of cases, and was associated with older age, male gender, higher Breslow thickness, ulceration, advanced clinical stage, and positive SLN status (all *p* < 0.01). However, only half of class 2 patients had ulcerated tumors and a quarter of them had a positive SLN biopsy. Mitotic rate, primary tumor location, and location of first recurrence were not associated with a class 2 profile.

**Table 1:** Demographic characteristics of 322 patients enrolled in two prospective registries and correlation with GEP Class

	All cases (n* = 322)	Class 1 (*n* = 248)	Class 2 (*n* = 74)	*p* value***
Age, median (range)	58 (18–87)	57 (18–87)	65 (23–85)	0.003
Gender				
Female	146 (45%)**	123 (50%)	23 (31%)	0.005
Male	176 (55%)	125 (50%)	51 (69%)	
Breslow thickness, median (range)	1.2 (0.2–12.0)	1.0 (0.2–7.0)	2.5 (0.4–12.0)	<0.001
Ulceration				
Absent	238 (74%)	204 (82%)	34 (46%)	<0.001
Present	58 (18%)	23 (9%)	35 (47%)	
Unknown	26 (8%)	21 (9%)	5 (7%)	
Mitotic rate				
≤1/mm^2^	222 (69%)	176 (71%)	46 (62%)	0.151
>1/mm^2^	100 (31%)	72 (29%)	28 (38%)	
Node status				
Negative	286 (89%)	228 (92%)	58 (78%)	0.007
Positive	36 (11%)	20 (8%)	16 (22%)	
Primary tumor location				
Extremity	178 (55%)	133 (54%)	45 (61%)	0.547
Head and neck	58 (18%)	46 (19%)	12 (16%)	
Trunk	86 (27%)	69 (28%)	17 (23%)	
AJCC stage				
None	3 (1%)	3 (1%)	0 (0%)	<0.001
I	1 (0%)	1 (0%)	0 (0%)	
IA	63 (20%)	62 (25%)	1 (1%)	
IB	145 (45%)	129 (52%)	16 (22%)	
IIA	39 (12%)	23 (9%)	16 (22%)	
IIB	26 (8%)	9 (4%)	17 (23%)	
IIC	8 (2%)	0 (0%)	8 (11%)	
III	30 (9%)	16 (6%)	14 (19%)	
IIIA	3 (1%)	3 (1%)	0 (0%)	
IIIB	3 (1%)	1 (0%)	2 (3%)	
IV	1 (0%)	1 (0%)	0 (0%)	
Location of first recurrence				
Nodal/regional	13	3	10	0.69
Distant	12	2	10	

*n = number of samples

**All percentages are reflective of the indicated group out of the total number of samples in that column

***p values reflect differences between class 1 and 2 patients and were calculated using chi-squared or *F* tests as appropriate

### Interim analysis of outcomes

At 1.5 years of follow-up, regional metastasis was the first site of recurrence in 52% (13 of 25) of cases, and 77% (10 of 13) of those were identified as high-risk class 2. Similarly, for the 12 patients for whom the site of first recurrence was a distant metastasis, 83% (10 of 12) were identified as class 2.

The GEP test was the most sensitive prognostic factor for all endpoints assessed, including SLN biopsy and ulceration, with a high-risk class 2 result showing 80% sensitivity for recurrences, 83% for distant metastases, and 73% for death (Table 2). Overall, recurrence rates were 2% (5 of 248) for class 1 versus 27% (20 of 74) for class 2 patients, distant metastasis rates were 1% (2 of 248) versus 14% (10 of 74), and overall survival rates were 1% (3 of 248) versus 11% (8 of 74), respectively.

**Table 2:** Outcomes in the 322-patient prospective cohort and correlation with GEP class, SLN status, and ulceration

	Recurrence (n* = 25)	Distant metastasis (*n* = 12)	Death (*n* = 11)
Class 1 (*n* = 248)	5 (2%)	2 (1%)	3 (1%)
Class 2 (*n* = 74)	20 (27%)	10 (14%)	8 (11%)
*p* value**	<0.0001	<0.0001	0.0001
SLN− (*n* = 286)	15 (5%)	6 (2%)	10 (3%)
SLN+ (*n* = 36)	10 (28%)	6 (17%)	1 (3%)
*p* value	<0.0001	<0.001	1
Ulceration absent (*n* = 264)	10 (4%)	3 (1%)	6 (2%)
Ulceration present (*n* = 58)	15 (26%)	9 (16%)	5 (9%)
*p* value	<0.0001	<0.0001	0.04

*n = number of samples

**p values reflect differences between high and low-risk groups for each prognostic factor (*χ* ^2^ test)

**Fig. 1:**
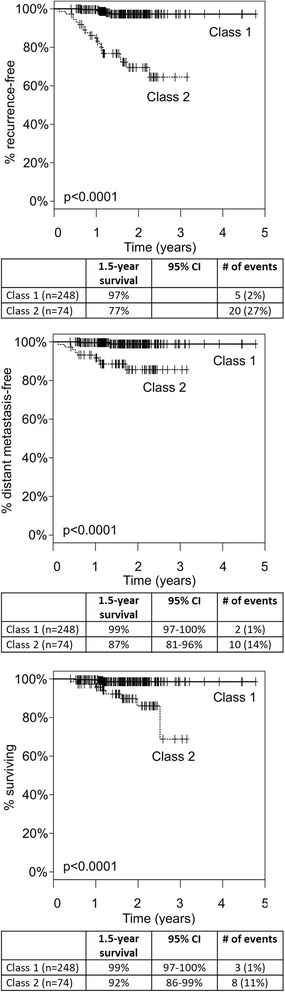
Kaplan-Meier analysis of RFS (top), DMFS (middle), and OS (bottom) by GEP class. Significant separation of class 1 and class 2 risk (*p* < 0.0001) is observed for each endpoint

Median recurrence event times for class 2 patients in prior retrospective studies is 1.1 years after diagnosis [12, 21, 22]. With a median follow-up time for non-event samples that exceeds 1.5 years for all endpoints, interim analysis of outcomes in this population is possible. The GEP test was significantly associated with recurrence (*p* < 0.001), distant metastasis (*p* < 0.001), and death (*p* < 0.001). The 1.5-year RFS, DMFS and OS rates for class 1 versus class 2 were 97% (95–100%) versus 77% (67–87%), 99% (97–100%) versus 87% (81–96%), and 99% (97%–100%) versus 92% (86–99%), respectively (Fig. 1). The GEP test was also significantly associated with each endpoint analyzed in the group of subjects with stage I or II melanoma (*n* = 282, Additional file 1: Figure S1).

Multivariate Cox regression modeling (*n* = 296 complete cases) showed that BT, mitotic rate, and GEP class were independent predictors of RFS outcome (Table 3; *p* < 0.01, BT – HR: 1.43 [95% confidence interval (CI) 1.18–1.73], mitotic rate – HR: 1.05 [95% CI 1.01–1.08], GEP – HR: 7.15 [95% CI 1.99–25.8]). For RFS, node status was not significant in these interim analyses at an alpha of 0.01 [*p* = 0.035, HR: 2.46 (95% CI 1.07–5.68)]. For DMFS and OS, only Breslow thickness was significant [*p* < 0.001, Additional file 2: Table S1].

**Table 3:** Multivariate Cox regression analysis of RFS

	HR (95% CI)	*p* value
Breslow thickness	1.43 (1.18–1.73)	0.001
GEP class 2	7.15 (1.99–25.8)	0.003
Mitotic rate	1.05 (1.01–1.08)	0.005
SLN positivity	2.46 (1.07–5.68)	0.035
Ulceration present	1.89 (0.75–4.72)	0.17

### Comparison to retrospective cohorts

**Fig. 2:**
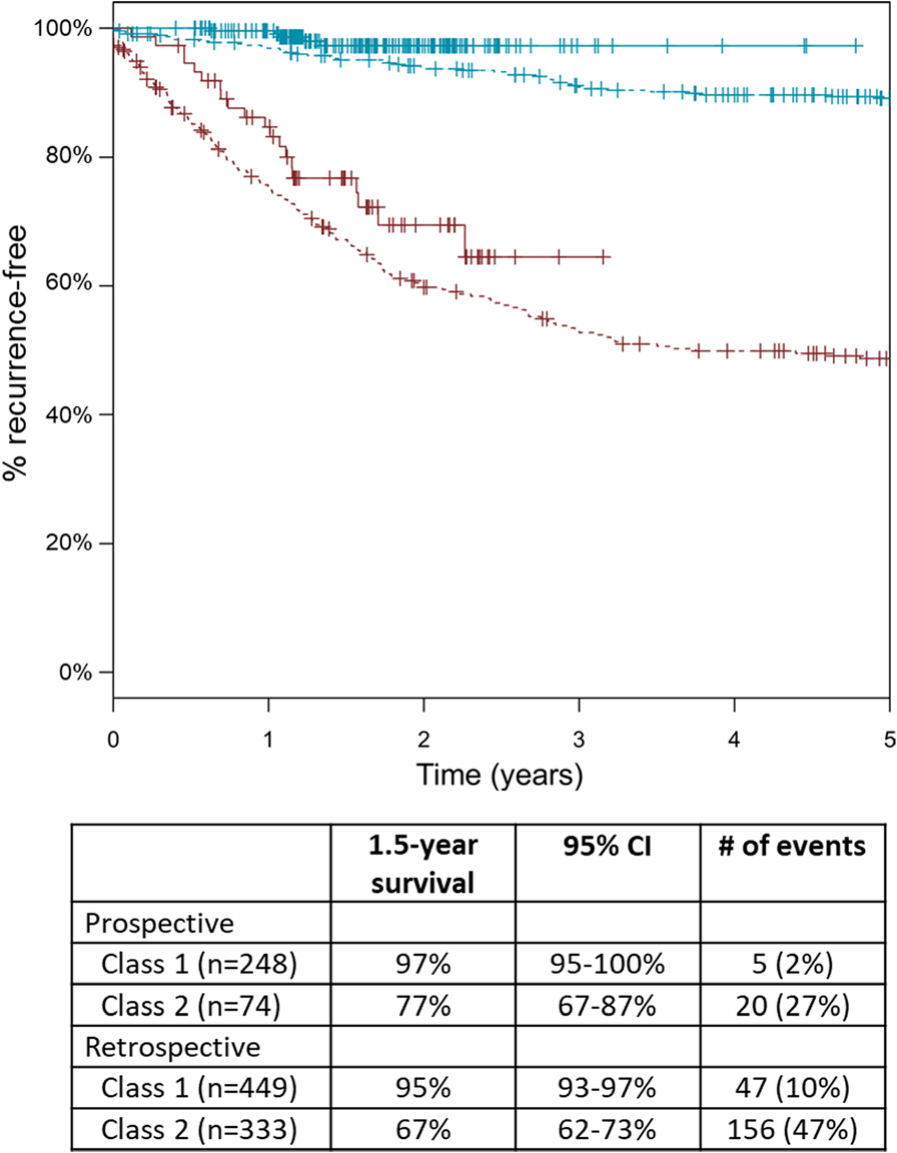
Kaplan-Meier recurrence-free survival curves for class 1 (blue) and class 2 (red) subjects in the prospective registry cohort (solid lines; *n* = 322) and collective retrospective studies (dashed lines; *n* = 782)

Table 4 summarizes the outcomes data for this prospective trial and previously reported retrospective analyses. The original report of the GEP test performance included 104 samples, with median follow-up time of 8.2 years for non-event samples and 1.7 years for samples with an event (Table 4) [12]. The 1.5-year RFS rates were 98% (95% CI 95–100%) for class 1 and 70% (95% CI 58–85%) for class 2. The most recent validation dataset included 523 stage I-III long-term follow-up cases [22]. In this dataset, median follow-up time for cases without an event was 7.2 years and median time to a recurrence event was 1.2 years. For all cumulative cases that have been reported in retrospective validations (total for all cases: *n* = 782), the median time to a recurrence event was 1.3 years (1.1 years for the class 2 cohort alone). The 1.5-year RFS rates were 95% (95% CI 93–97%) for class 1 cases and 67% (95% CI 62–73%) for class 2 cases. While the median follow-up time is shorter for this prospective analysis, with 1.5 years of follow-up for cases without an event and 1.0 year for cases with an event, the observed RFS rates of 97% for class 1 and 77% for class 2 are consistent with 1.5-year event rates in prior retrospective datasets (Table 4, Fig. 2).

**Table 4:** Summary table of RFS rates observed in the prospective registry cohort and retrospective archival studies

Study	*n*	Non-event follow-up time	Event time	Class 1 1.5-year RFS	Class 2 1.5-year RFS
		Median, years	Median, years	Percent (95% CI*)	Percent (95% CI)
Initial validation (Gerami, 2015)	104	8.2	1.7	98% (95–100%)	70% (58–85%)
Independent validation	523	7.2	1.2	95% (92–97%)	67% (61–74%)
All retrospective studies to date	782	6.9	1.3	95% (93–97%)	67% (62–73%)
Current study	322	1.5	1.0	97% (95–100%)	77% (67–87%)

*CI confidence interval

## Discussion

An important unmet clinical need in melanoma is the accurate identification of early-stage patients who harbor a higher risk of developing advanced disease. While Breslow thickness and ulceration are strongly associated with metastasis and outcome, and sentinel lymph node status is a proven prognostic tool that identifies a subset of the patients with high-risk disease, two of three patients who die from melanoma are originally diagnosed with stage I or II tumors [24]. As these clinicopathologic staging features are used to drive management decisions, molecular tools that supplement and improve current methods of prognostication should have significant clinical impact.

To improve upon the identification of high-risk stage I and II patients, a gene expression profile test was developed to provide prognostic information based on the expression of 31 genes in the primary melanoma tumor [12]. A limitation of this interim analysis could be the impact of the short follow-up time. However, consistent with previous reports, the GEP test was a significant predictor of RFS, DMFS, and OS, in both the larger cohort of 322 patients (Fig. 1), and in 282 stage I and II CM cases (Additional file 1: Figure S1). The risk of metastasis and death was significantly higher for patients with a class 2 result compared to class 1 patients. The GEP accurately identified 80% of the recurrences detected during the study, and only 2% of the patients who were predicted to be low risk (class 1) by the test developed recurrent disease. Although multivariate analysis showed BT to be the only independent prognostic factor for DMFS and OS in this cohort, this is likely to be due to the limited number of events for these two outcomes. In all prior studies the GEP has shown independent prognostic value along with Breslow thickness and SLN status.

In this study, 83% (10 of 12) of patients who developed distant metastases were identified as high risk by the GEP test, compared to only 50% (6 of 12) who had a SLN-positive result. Additionally, we found that the median time to recurrence for those who developed advanced disease was only 1 year. National melanoma guidelines specify that patient management should be tailored to an individual’s probability of recurrence. Frequent follow-up and intensified surveillance with imaging is recommended for patients with high-risk clinicopathologic features, while management of low-risk patients is generally restricted to clinical follow-up at 3–12 month intervals [25]. A growing body of evidence supports cross-sectional radiographic imaging as the most effective method for detecting asymptomatic distant metastatic disease in patients with stage II and III melanoma [7, 10, 11]. In this study, 6 of 12 patients with distant metastasis were originally diagnosed with stage II disease. Of those, five were identified as class 2, including two patients with stage IIA tumors. While direct evidence of a benefit from surveillance has not been published, considering the rapid time to event observed in this interim analysis, and the accuracy of risk prediction by the GEP test, increased surveillance with imaging for class 2 patients might be useful, especially in those patients who would not be offered surveillance options based on stage.

The advantages of molecular testing for enhanced prognosis are well documented for other diseases, including breast cancer and ocular melanoma [26, 27]. Consistent with previous studies, the results from this prospective analysis indicate that the 31-gene GEP test for melanoma, in combination with standard clinicopathologic factors, can strengthen risk determination and improve patient management. Better risk prediction is particularly critical when considering the recent advances in therapeutics for melanoma and the evidence supporting better efficacy of contemporary therapies if treatment is administered when tumor burden is low [28, 29].

## Conclusions

This constitutes the first report of performance for the prognostic 31-gene GEP test in a prospective population of patients with cutaneous melanoma. In concordance with prior retrospective studies, the test showed robust ability to predict recurrence, distant metastasis and death. While this report encompasses only an interim analysis and, therefore, it is expected that additional events will accrue in this population, the strong statistical association with outcomes even at this early time point provides assurance of the test’s prognostic value.

## Additional files

Additional file 1: Figure S1. Survival outcomes for stage I/II patients with molecular classification by the 31-gene expression profile test. A) Recurrence-free survival, B) distant metastasis-free survival, and C) overall survival for Class 1 and Class 2 subjects with stage I or stage II disease (*n* = 282). (TIFF 192 kb)

Additional file 2: Table S1. Cox regression analysis for distant metastasis-free (DMFS) and overall survival (OS) in the 322-subject cohort. (DOCX 12 kb)

## Abbreviations

AJCC: American Joint Committee on Cancer
ANOVA: Analysis of variance
CAP/CLIA: College of American Pathologists/Clinical Laboratory Improvement Amendments
CI: Confidence interval
CM: Cutaneous melanoma
DMFS: Distant metastasis-free survival
GEP: Gene expression profile
OS: Overall survival
RFS: Recurrence-free survival
SEER: Surveillance, Epidemiology, and End Results
SLN: Sentinel lymph node
