# Erratum to: Interim analysis of survival in a prospective, multi-center registry cohort of cutaneous melanoma tested with a prognostic 31-gene expression profile test

**DOI:** 10.1186/s13045-017-0524-x

**Published:** 2017-10-05

**Authors:** Eddy C. Hsueh, James R. DeBloom, Jonathan Lee, Jeffrey J. Sussman, Kyle R. Covington, Brooke Middlebrook, Clare Johnson, Robert W. Cook, Craig L. Slingluff, Kelly M. McMasters

**Affiliations:** 1 Department of Surgery, St. Louis University, St. Louis, MO USA; 2 South Carolina Skin Cancer Center, Greenville, SC USA; 3 Northside Melanoma and Sarcoma Specialists of Georgia, Atlanta, GA USA; 4 Department of Surgery, University of Cincinnati Cancer Institute, Cincinnati, OH USA; 5 Castle Biosciences, Inc., 820 S. Friendswood Drive Suite 201, Friendswood, TX USA; 6 Department of Surgery and Cancer Center, University of Virginia School of Medicine, Charlottesville, VA USA; 7 Department of Surgical Oncology, James Graham Brown Cancer Center, University of Louisville School of Medicine, Louisville, KY USA

## Erratum

The original article [1] contained an error in Fig. 1 mistakenly carried forward by the Production team handling this article whereby the values denoting the 95% Confidence Intervals in the uppermost table had been omitted.

**Fig. 1:**
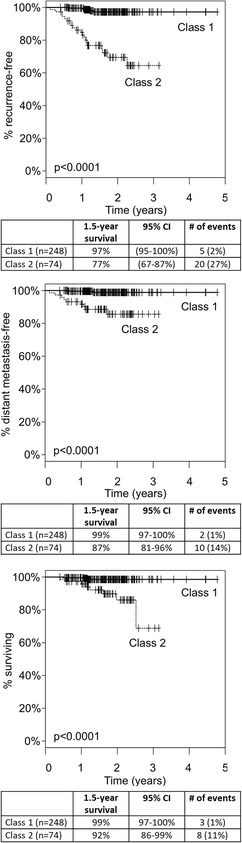
Kaplan-Meier analysis of RFS (top), DMFS (middle), and OS (bottom) by GEP class. Significant separation of class 1 and class 2 risk (*p* < 0.0001) is observed for each endpoint. Solid line: class 1; dashed line: 2

This error has now been corrected and the values returned.
