# Supplementary figures and images for: Interim analysis of survival in a prospective, multi-center registry cohort of cutaneous melanoma tested with a prognostic 31-gene expression profile test
Source: J Hematol Oncol. 2017 Aug 29;10:152. doi: 10.1186/s13045-017-0520-1 (PMC5576286; doi:10.1186/s13045-017-0520-1)

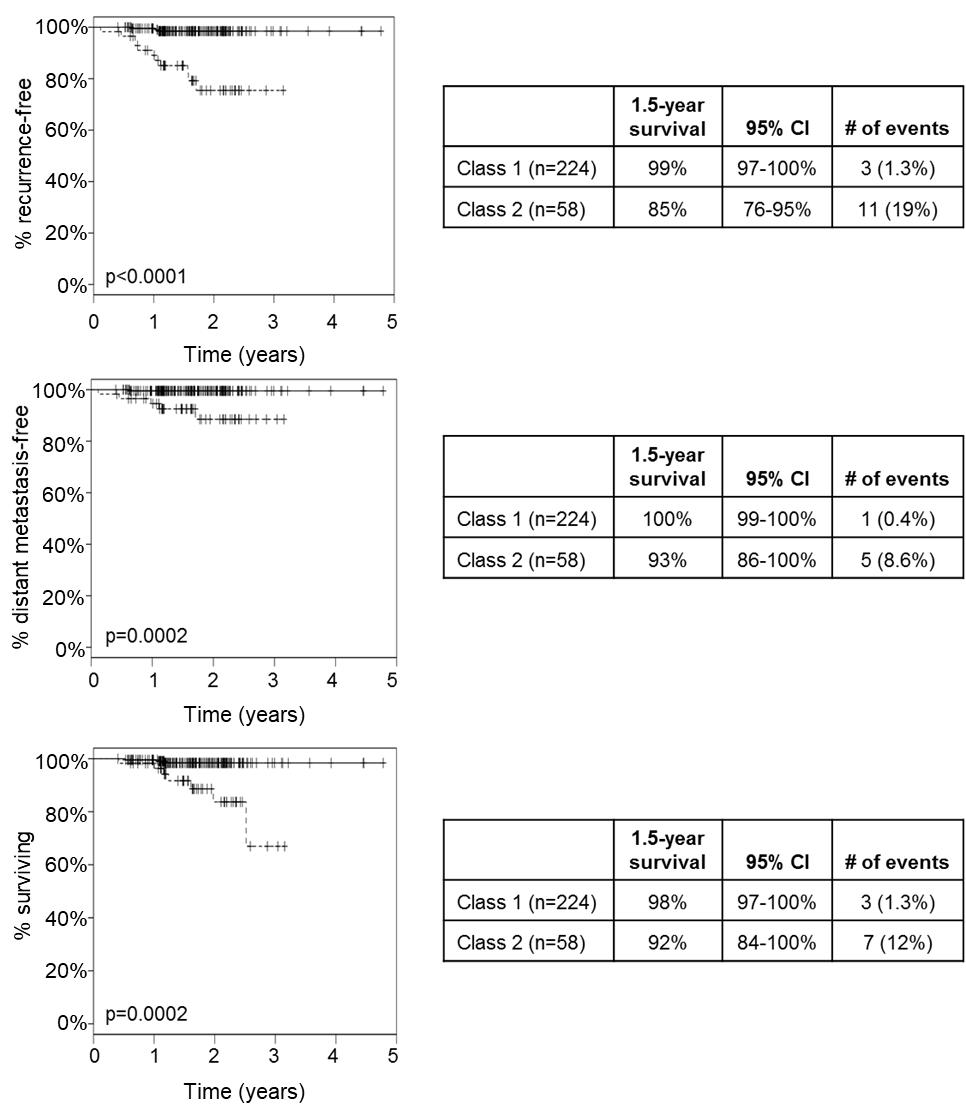

Supplement: Supplementary file 1 — Survival outcomes for stage I/II patients with molecular classification by the 31-gene expression profile test. A) Recurrence-free survival, B) distant metastasis-free survival, and C) overall survival for Class 1 and Class 2 subjects with stage I or stage II disease (n = 282). (TIFF 192 kb) [file 13045_2017_520_MOESM1_ESM.tif]
