# Supplementary material for: Interim analysis of survival in a prospective, multi-center registry cohort of cutaneous melanoma tested with a prognostic 31-gene expression profile test
Source: J Hematol Oncol. 2017 Aug 29;10:152. doi: 10.1186/s13045-017-0520-1 (PMC5576286; doi:10.1186/s13045-017-0520-1)
Supplement: Supplementary file 2 — Cox regression analysis for distant metastasis-free (DMFS) and overall survival (OS) in the 322-subject cohort. (DOCX 12 kb) [file 13045_2017_520_MOESM2_ESM.docx]

**Table S1.** Cox regression analysis for distant metastasis-free (DMFS) and overall survival (OS) in the 322-subject cohort.

| **DMFS** | **HR (95% CI)** | **p value** |
| --- | --- | --- |
| Mitotic rate | 1.03 (0.97-1.09) | 0.34 |
| Ulceration present | 3.12 (0.68-14.4) | 0.14 |
| Breslow thickness | 1.58 (1.25-1.98) | 0.0001 |
| SLN positivity | 3.18 (0.99-10.2) | 0.051 |
| GEP Class 2 | 2.98 (0.47-19.1) | 0.25 |
| **OS** | **HR (95% CI)** | **p value** |
| Mitotic rate | 0.98 (0.81-1.18) | 0.79 |
| Ulceration present | 1.04 (0.24-4.48) | 0.95 |
| Breslow thickness | 1.5 (1.19-1.89) | 0.0005 |
| SLN positivity | 0.3 (0.03-2.55) | 0.27 |
| GEP Class 2 | 3.73 (0.77-18.1) | 0.10 |

*CI, confidence interval
